# Use of Pyrolysis–Gas Chromatography/Mass Spectrometry as a Tool to Study the Natural Variation in Biopolymers in Different Tissues of Economically Important European Softwood Species

**DOI:** 10.3390/polym15214270

**Published:** 2023-10-30

**Authors:** David Hentges, Philippe Gérardin, Pierre Vinchelin, Stéphane Dumarçay

**Affiliations:** Faculté des Sciences et Technologies, Université de Lorraine, Inrae, Lermab, F-54000 Nancy, France; hentges.david@yahoo.de (D.H.); philippe.gerardin@univ-lorraine.fr (P.G.); pierre.vinchelin@univ-lorraine.fr (P.V.)

**Keywords:** pyrolysis–gas chromatography, Py-lignin, holocellulose, PCA, markers

## Abstract

Intraspecific macromolecule variation in stemwood, knotwood, and branchwood was studied using analytical pyrolysis with the intention of introducing a rapid working method to assess the variance in lignin content using analytical pyrolysis and highlight variability markers. The study was performed on *Picea abies*, *Abies alba*, and *Pseudotsuga menziesii*. Lignin determined via analytical pyrolysis–GC/MS (Py-lignin) can be used to identify variations in lignin content, compared to using classical Klason lignin values as a reference method for lignin determination, which requires a correction factor. Principal component analysis (PCA) was performed to identify biopolymer pyrolysis product markers for different species, tissues, or heights that could help highlight structural differences. Douglas fir was differentiated from spruce and silver fir in the levoglucosan amount. Guaiacol was more present in spruce wood, and creosol was more present in Douglas fir. Knotwood was structurally close to stemwood in spruce and silver fir, but there was a clear transition between stemwood and branchwood tissue in Douglas fir. Knotwood was differentiated by higher furan compounds. Branchwood was clearly separate from stemwood and knotwood and presented the same markers as compression wood in the form of phenylpropanoid lignins (H-lignin) as well as isoeugenol and vinyl guaiacol, the two most produced lignin pyrolysis products.

## 1. Introduction

Lignin is a phenolic macromolecule and the second most abundant biopolymer on Earth after cellulose. As such, it has garnered much interest as it can be exploited for a large range of possibilities, such as biofuel feedstocks and value-added chemicals [1].

Knowing how much of the biopolymer is available from different biomasses has led to the development of different techniques that assess the quantity of lignin directly or indirectly. Standard methods for assessing wood polymers are based on wet chemistry, including the Klason method for lignin, sodium chlorite, and NaOH, or the Kürschner Hoffer method for holocellulose and cellulose. 

Numerous methods such as mid-infrared and near-infrared spectroscopy (MIR and NIR), nuclear magnetic resonance (NMR), high-performance liquid chromatography (HPLC), gas chromatography (GC), size exclusion chromatography (SEC), and many more have been used to determine the lignin content in wood [2]. The authors compared the advantages and disadvantages of numerous techniques and concluded that there is no superlative analytical method that can be used to determine lignin content in wood, but they highlighted the numerous advantages of pyrolysis coupled with gas chromatography and mass spectrometry (Py-GC/MS), as it usually requires around 1 mg and only needs pre-treatment, corresponding to the reduction of wood to sawdust and the removal of extractives. 

Lignin determined via Py-GC/MS using all the pyrolysis products from guaiacyl (G) units as well as syringyl (S) units in hardwood, or even *p*-hydroxyphenyl (H) units, is called Py-lignin. It has been used as an indicator instead of Klason lignin and has been confirmed to be a suitable method for different species, such as hardwoods like eucalyptus and softwoods like spruce, maritime pine, and larch [3,4,5,6,7].

However, a high number of pyrolysis products are obtained from each sample, which potentially requires multivariate statistical techniques such as PCA to compare structural data between similar samples to highlight sharp differences corresponding to small variations in the chemical composition.

Indeed, beyond the well-known natural variation in wood polymers between different wood species, evaluation of, e.g., the variation in lignin from the base of the trunk to the top of the crown could be of interest in the fields of metabolism studies, wood pulping or other valorizations. 

The objectives of this study were multiple. The first was the study of the variation in wood polymers according to the height of three economically significant softwood species, namely *Picea abies* [8,9,10], *Abies alba* [11,12,13], and *Pseudotsuga menziesii* [14,15], as well as along their branches using pyrolysis–gas chromatography coupled with mass spectrometry. The highlighted works portray an incomplete picture of the chemical variation in wood polymers in *Abies alba* and *Pseudotsuga menziesii* in particular. To our knowledge, no studies comparing the pyrolysis products of these species have been conducted before, as demonstrated by the scarce number of publications on the subject. 

The second objective was to analyze the potential structural differences through markers from lignin and holocellulose pyrolysis products.

## 2. Materials and Methods

Samples of stemwood, knotwood, and branchwood were used. Table 1 contains the necessary information on the trees from a subset of the samples used in our previous publication [16]. 

Thinning is the process of removing trees adjacent to the sample of interest to improve its growth rate thanks to weaker competition. Branchwood was supplied from the same trees, but no special attention was given to their original height.

Three different samples from each tree were taken to represent three different heights. The bottom corresponds to 130 cm for stemwood or to the lowest detected live knot, depending on the tree in the case of knotwood. The top is defined where the tree has a maximum diameter of 10 cm for stemwood and for knotwood, the highest sample that had enough sawdust to perform all tests. The middle corresponds to samples ideally equidistant from the top and bottom samples. 

For branchwood, five samples were taken from each branch and labeled according to their distance to the trunk in cm: 0, 25, 50, 100, and 250. One branch was studied for every species.

### 2.1. Preparation of Wood Samples

The knotwood was roughly separated from the stemwood at first. In a second step, it was separated from the remaining stemwood surrounding it with a chisel. The distinction between the tissues was made using the youngest growth ring from the knot. Some knots were difficult to distinguish between knotwood and stemwood, which resulted in partial loss of the outer ring. No attempt to separate different parts like side wood, opposite wood, or compression wood [17] of the knot was made as the samples were primarily prepared for tests prior to this work. 

Stemwood and branchwood were obtained in the form of disks of 1 cm after removal of the bark.

The stemwood, knotwood, and branchwood were milled in a Fritsch Pulverisette 9 mill (Fritsch, Idar Oberstein, Germany, 2011) at 1100 rpm for around 2 min. The collected wood was oven-dried at 103 °C for at least 8 h, after which the samples were extracted in a Soxhlet apparatus with toluene/ethanol (2/1 *v*/*v*) followed by an extraction with 96% ethanol, both for at least 8 h.

After extraction, the sawdust was passed through 2 different sieves, 0.1 mm and 0.4 mm, so that 2 fractions were recovered for different purposes. The first sample size (<0.1 mm) was used for Py-GC/MS and the latter (0.1–0.4 mm) for wet chemical analysis because too-small samples may result in underestimating results in wet chemical analysis (Laboratory Analytical Procedure, 2008). Furthermore, using a precise amount for pyrolysis is easier when using fine sawdust. All measurements were performed in duplicate.

### 2.2. Flash Pyrolysis

The following program was designed according to literature results and internal testing. The final temperatures and amount used were chosen because they provided a strong, unsaturated, and distinct signal of every compound of interest. Lower temperatures did not provide enough compounds of higher molecular weights and higher temperatures and had less variety. 

Around 0.7 mg of extracted sawdust was introduced into a small quartz tube, and both ends were filled with quartz wool. The tube was inserted in a platinum filament coil of a Pyroprobe 5200 (CDS analytical, Oxford, PA, USA). The pyrolysis was performed at 550 °C for 5 s. The pyrolysate was transferred through a transfer column heated at 280 °C into an injector maintained at the same temperature and connected to a GC/MS (Perkin Elmer, Clarus 580 Gas Chromatograph/Perkin Elmer, Clarus 500 Mass Spectrometer, Waltham, MA, USA) with a split of 75 mL/min. The oven is heated at 50 °C for 5 min, followed by a ramp-up in temperature of 6 °C/min up to 280 °C. This final temperature was held for 6.67 min for a total experiment duration of 50 min. The column used was a DB-1701 (60 m × 0.5 mm × 0.5 µm, Agilent J&W, Santa Clara, CA, USA). The GC/MS interface temperature was held at 280 °C and the ionization energy and temperature at 70 eV and 250 °C, respectively. The MS range was set from 28 to 300 *m*/*z*.

Comparison was made with the NIST database as well as with the literature in order to identify the detected compounds [3,9,18,19,20].

### 2.3. Wet Chemical Analysis

#### 2.3.1. Lignin Content

The lignin content of all samples was determined using the Klason lignin method following the procedure of LAP (Laboratory Analytical Procedure, 2008) with some slight modifications. The reaction was conducted using 300 mg of sawdust in 100 mL flasks with caps. Although the softwood species usually have a negligible amount of acid-soluble lignin (ASL), the measurement was taken regardless to check if it changed significantly between samples. The acid-soluble lignin was determined using an extinction coefficient index of 110 L/(g*cm) at 205 nm. Therefore, the reported lignin corresponds to the sum of the insoluble Klason lignin with the acid-soluble lignin. 

#### 2.3.2. Holocellulose Content

Exactly ca. 500 mg of sawdust and 30 mL of distilled water were introduced into a 250 mL flask, which was then heated to 75 °C and stirred with a magnet. For the next 7 h, 0.1 mL of acetic acid and 2 mL of aqueous sodium chlorite (15%) were added to the reaction every hour. The mixture was then filtered on a Büchner funnel and washed with distilled water and ethanol until the yellow coloring disappeared. The holocellulose was dried at 103 °C for at least 8 h and weighed [21]. The holocellulose obtained is stated as a percentage of extractive-free wood.

#### 2.3.3. α-Cellulose Content

Alpha-cellulose content determination was adapted from Rowell [22]. All the holocellulose gained from the previous section was inserted into a 250 mL flask and mixed with 10 mL of a 17.5% NaOH solution. Every 5 min for the next 15 min, 5 mL of the NaOH solution was added to the stirred slurry. The mixture was left stirring for 30 min at room temperature. It was then diluted with 33 mL of distilled water and left to rest for 1 h. The solution containing the α-cellulose was inserted into a centrifuge for 5 min at 4400 rpm. The supernatant containing the solubilized hemicelluloses was eliminated, and the cellulose residue was filtered on a Büchner funnel, washed with water, followed by 15 mL of acetic acid (10%) and water again. The α-cellulose was dried at 103 °C for at least 8 h and then weighed. The α-cellulose obtained is stated as a percentage of extractive-free wood.

### 2.4. Peak Integration and Statistical Analysis

All the results obtained from wet chemical analysis were subjected to an analysis of variance (ANOVA) with significant differences between the different heights and trees from the same species determined using the Tukey HSD method at a *p*-value threshold of 0.05. The Klason lignin and cellulose samples of each species were tested for normal distribution as well as homogeneity of variances to verify the independence of samples. 

Peak integration was performed with Openchrom 1.5.0, an open-source piece of software designed for Py-GC/MS data. The Py-lignin was determined as the ratio of the sum of the area of lignin and all pyrolysis products multiplied by 100. 

Finally, Principal Component Analysis (PCA) was used to describe the patterns of covariation between height, compartment, and species. This was carried out with RStudio using normalized data of all the polysaccharides and lignin pyrolysis product peaks. No data correction factor, like Savitzky–Golay smoothing or baseline removal, was performed as it was observed that the former could change the Py-lignin results by up to 4%.

## 3. Results

### 3.1. Py-Lignin

#### 3.1.1. Stemwood and Knotwood

**Figure 1:**
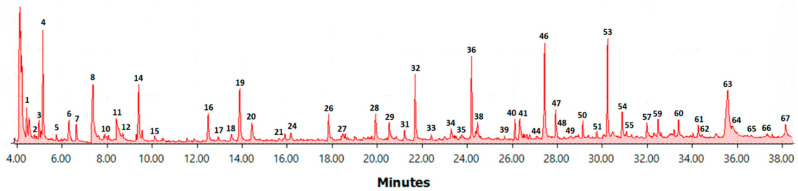
Spruce stemwood pyrogram. The compounds belonging to the labeled peaks are listed in Table 2.

A typical pyrogram of spruce stemwood is presented in Figure 1.

The spruce stemwood chromatogram (Figure 1) broadly represents all the samples used in this paper. All the major phenolic peaks were used in determining Py-lignin with a few exceptions, such as the first peak (CO_2_), the peaks of toluene and ethanol since they might be residuals from the extraction process, as well as peaks that represent less than 0.01% of total peak area. All peaks not belonging to the phenolic compound family were classified as carbohydrate derivatives to simplify the method. Overlapping peaks were taken into account if all the identified compounds belonged to the same family.

A summary of all peaks with their retention time, compound family, main MS fragments, and area percentage range can be found in Table 2.

**Figure 2:**
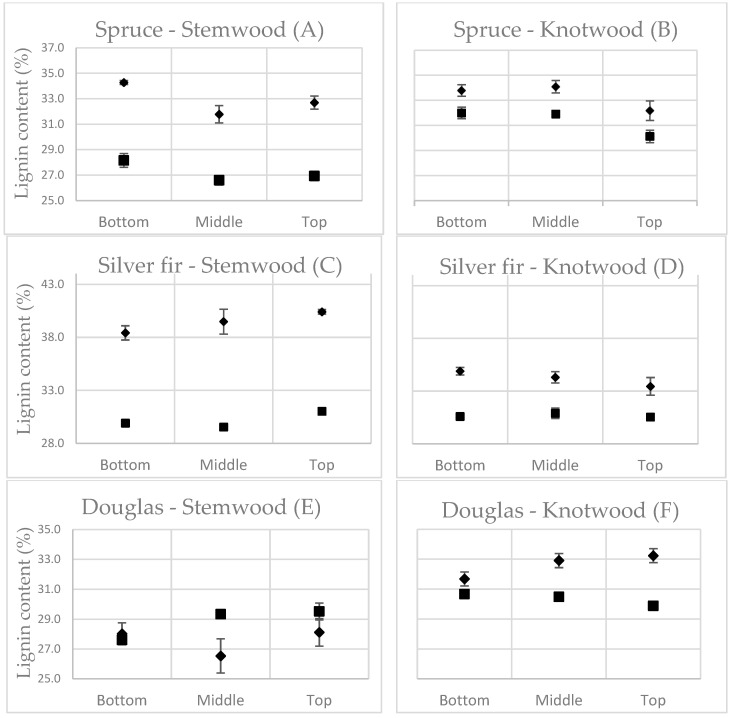
Klason lignin (filled square) and Py-lignin (filled diamond) comparison for stemwood and knotwood samples.

The Py-lignin contents are depicted with Klason lignin in Figure 2. The bottom, middle, and top Klason lignin and Py-lignin values are the average values of all 4 trees. All un-thinned and thinned samples are averaged together in the presented values because no significant difference was found, as elaborated later in Table 3. Error brackets are the standard deviation of the average between duplicates to compare the precision of both methods.

The standard deviation for Py-lignin was 0.89 for spruce, 0.79 for silver fir, 1.55 for Douglas fir, and 0.66, 0.42, and 0.50 for Klason lignin, respectively, which was slightly above the reported values from Alves et al. [3] for spruce.

For stemwood, the bottom, middle, and top variables were at approximately the same relative height for all species. For knotwood, the relative height of knots varied more broadly concerning the bottom position, which might explain higher variances. Since juvenile wood, sapwood, and heartwood were mixed, our samples were to be considered an average of all three. Naturally, depending on the height, there was a greater relative quantity of juvenile and sapwood. Though this might explain some higher or lower quantities of Klason lignin, it only marginally affected Py-lignin content.

The comparison of Klason lignin to Py-lignin showed differences according to species and wood tissue. A different correction factor equal to the difference of bottom, middle, and top averages of Klason lignin and Py-lignin was needed. The differences were as follows: (A) 5.7%, (B) 2%, (C) 9.3%, (D) 3.5%, (E) −1.3%, (F) 2.3%.

Differences between heartwood and knotwood were expected since the latter has a higher content of compression wood [17,23].

When considering the corrected Py-lignin values with the correction factors, they were within the error brackets for all the spruce and silver fir samples, validating Py-lignin as an effective variation marker in lignin in stemwood as well as in knotwood. Douglas fir was an exception to this rule. The trees labeled 1 and 3 each had a strongly different Py-lignin value in the bottom sample compared to Klason lignin and all other Douglas fir samples’ Py-lignin amounts. The reasons for these differences are not fully understood, but it seems that Douglas fir has potential structures impacting pyrolysis mechanisms. Multiple re-injections of the samples resulting in the same results excluded pyrolysis problems. One possible reason might be the presence of inorganic salts [24,25,26,27] decreasing holocellulose content, though this is unlikely because ethanol extraction should eliminate most, if not all, of them.

Numerous factors influencing pyrolysis could explain the difference between Klason lignin and Py-lignin depending on tissue and species. Lignin content, as well as cellulose and hemicellulose contents, react differently in different proportions [28,29], though the authors discussed the important factor that lignin-carbohydrate-complexes (LCC) also play in the formation of important products such as levoglucosan. LCCs take into account all the lignin moieties in softwoods [30,31], and different hemicellulose compositions lead to differing pyrolytic results [32]. Coniferous species are almost exclusively composed of G-lignin. This enables many possibilities of cross-linking between lignin moieties, which vary depending on the species. Other factors, such as free phenolic structures, have a lower redox potential, producing different structures by pyrolysis [33,34].

All those factors highlight the importance that the Py-lignin values of every species and tissue need to be compared to Klason lignin beforehand in order to use this method effectively since it currently remains the commonly admitted standard.

By comparison with Alves et al. [3,7], too many variables for data acquisition were different for it to be possible to discuss the difference in Py-lignin content, most notably no FID (by measuring with TIC ranging from 28 to 300, we ignored the smaller fragments, which are taken into account for peak representation with FID), a different pyrolysis temperature and a different sample quantity. They observed in their works that the higher the Klason lignin content grew, the more Py-lignin was underestimated, but our samples revealed only differences between tissues, which seemed more influenced by structural characteristics such as density, crystallinity, etc.

##### Wet Chemical Analysis

As well as the Klason lignin presented along with Py-lignin, other well-established methods have been performed. The results of wet chemical analysis tests are presented in Table 3. The polymer contents are presented with the standard variation between all 4 trees.

Klason lignin and cellulose contents for stemwood were in the range of expected values for *Picea abies* [35,36], *Abies alba* [37,38], and *Pseudotsuga menziesii* [14]. Studies exploring the longitudinal variability of lignin in different species are scarce [35,39] and have not yet been performed extensively on silver fir, spruce, and Douglas fir.

The cellulose content variation was harder to discuss, as it seemed to vary more greatly between different trees, and no specific trend could be identified apart from usually being in the opposite manner to Klason lignin. Since holocellulose varied the same way as cellulose everywhere, hemicelluloses varied the same way by extension. For cellulose, the content was, on average, lower in knotwood compared to stemwood.

An attempt was made to use the α-cellulose data to try to estimate the cellulose or hemicellulose contents using Py-GC/MS, but like some previous works, we were unable to discriminate the pyrolysis products of both, using either all products or specific markers. Despite the fact that some markers were discussed by Lourenco et al. [40] like 1,4-anhydroxylopyranose, 1,5-anhydro-4-deoxy-pent-1-en-3-ulose, 4-hydroxy-5,6-dihydro-(2H)-pyran-2-one, furfural or levoglucosan to distinguish between hemicelluloses and cellulose, the use of these markers failed to emulate the results the same way that we obtained them for Py-lignin.

##### Statistical Analysis

Every different height and tree for a given compartment and species was subjected to an ANOVA analysis, with the results grouped in Table 4.

In general, there seemed to be no species or type of wood featuring a significant difference in lignin or cellulose contents according to height.

Some interactions between the different heights had a *p*-value lower than 0.05, which was usually explained by a punctual increase or decrease of a given polymer in a single tree. For example, the significant difference observed for silver fir stemwood was due to one tree having 34% lignin at the top, likely due to the formation of compression wood. There was no instance where 2 trees of the same modality were significantly different from the two trees of the opposite modality.

It is known that thinning enhances the growth rate of all remaining trees and increases the volume of the stand, but it can have negative influences on the physical properties of merchantable wood [41]. Tests such as those of Jyske et al. [42] have been performed to study the effect of thinning on chemical structures, which concluded with an absence of significant variation in tracheid properties and wood chemistry between 4 different thinning treatments. These results are mirrored in this paper. Thinning does not seem to affect wood chemical properties along the stem in stemwood or knotwood.

#### 3.1.2. Branchwood

**Figure 3:**
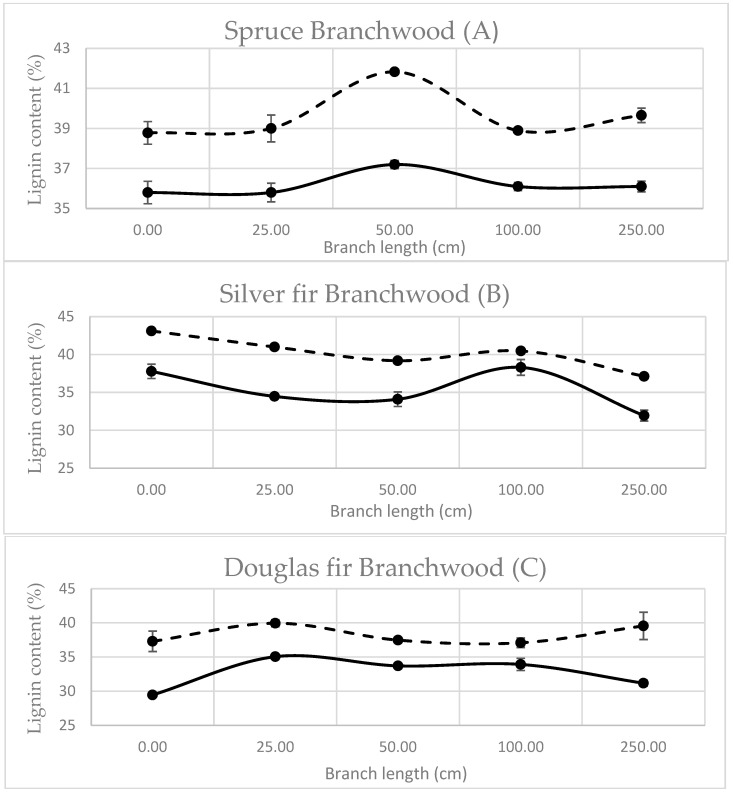
Klason lignin (solid line) and Py-lignin (dashed line) of branchwood samples.

Comparatively to compression wood at the base of the branch, branchwood has not been extensively studied in the literature because, usually, it is mostly considered non-recoverable and left as residual wood. To increase branchwood added value, it seemed important to gather more information on branches. Comparisons of Klason lignin and Py-lignin along the branches of spruce, silver fir, and Douglas fir are presented in Figure 3.

Several elements stood out: Py-lignin followed the same curve tendency along the branch as Klason lignin for all three species, apart from 250 cm in the Douglas fir branch. As with knotwood and stemwood, Douglas fir behaved the most erratically in its Py-lignin. The difference between Py-lignin and Klason lignin was 3.4% for spruce (A), 4.8% for silver fir (B), and 5.6% for Douglas fir (C). There did not seem to be a particular pattern to lignin content in branchwood apart from spruce. Since branchwood is mostly compression wood, whose growth is induced to keep the branch straight, there might be environmental factors, such as wind, animals, etc., that influence it differently along the branch to form different volumes of compression wood [43]. The differences between Py-lignin and Klason lignin could be taken as a correction factor for every sample apart from Douglas samples, as unknown irregularities seemed to tamper with pyrolysis results.

Knotwood and branchwood were different compared to stemwood as they both had a larger content of compression wood, which contains a higher lignin percentage [17,44] that can be as high as 40% and with a cellulose content that can be as low as 30%.

Lignin content along the branch did not seem to follow a clear scheme and might be dependent on the stress exerted at the specific spots along the branch, which induced the production of compression wood. It is known that thinning increases the volume of both branches and knots, but it does not seem to affect the wood composition significantly [45].

### 3.2. Principal Component Analysis

For every PCA presented, not all loadings are discussed, as only the most important differences are elaborated upon. The ellipses are arbitrarily drawn to include a maximum of points. Presented PCAs are biplots containing loadings as black dots with the labels explained in Table 3 and their labels and the samples as colored symbols. The main goal of the PCA was to approximate loadings with a species or tissue.

All samples of knotwood, heartwood, and branchwood of all Douglas fir, silver fir, and spruce were used and compared according to the indicated modalities. For more clarity, we chose to compare different PCAs to each other rather than plot all the points on a single figure to avoid a convoluted one.

#### 3.2.1. Differences between Species

**Figure 4:**
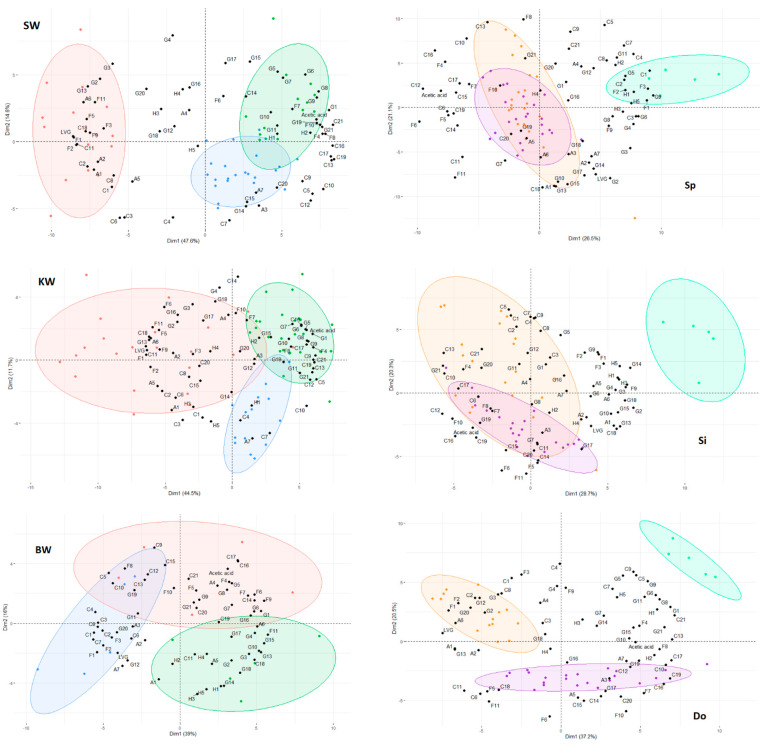
Principle component analysis of different tissues and species: (SW) stemwood, (KW) knotwood, (BW) branchwood, (Sp) spruce, (Si) silver fir, (Do) Douglas fir. Blue dots correspond to spruce, green dots to silver fir, red dots to Douglas fir, teal dots correspond to branchwood, orange dots to stemwood and purple dots to knotwood. Ellipse color corresponds to the same colored dots.

Figure 4 illustrates the PCA results between different species of stemwood, knotwood, and branchwood.

We chose to present different wood tissues and species separately because the similarity between silver fir and spruce, as well as their stemwood and knotwood, resulted in a poorly readable PCA. In this way, differences between the two species and Douglas fir, as well as between the different tissues, were clearly seen. PC1 is labeled as Dim1 (Dimension 1) and PC2 as Dim2 (Dimension 2).

Table 5 presents loadings that are re-occurring for certain tissues by comparing SW-KW. SW had an explained variance of 62.2% (PC1: 47.6%, PC2: 14.6%), KW a variance of 56.2% (PC1: 44.5%, PC2: 11.7%), and BW a variance of 55% (PC1: 39.0%, PC2: 16.0%). PCA dimensions 3, 4, and 5 were checked (not shown) to better classify each loading. Loadings not shown in the table had no specific tendency in positioning on the PCA. Spruce and silver fir samples varied less than Douglas fir samples, as demonstrated by the size of the clusters of the latter. The comparison of branchwood between different species (BW) was not considered in this table since the samples were more homogeneous. Work on more branches would need to be done to better distinguish them.

Of the four largest lignin peaks, trans-isoeugenol (G9) and *p*-vinylguaiacol (G5) were found in silver fir and branchwood, two of which had the highest Py-lignin amount. These two seemed to always correlate with higher lignin content, as they were the highest in quantity. Guaiacol and creosol, on the other hand, varied more, with this first compound being more present in spruce and the former compound more in Douglas fir.

According to PCA SW and KW, it seemed that levoglucosan (LVG) is characteristic of Douglas chemical composition in heartwood and knotwood. For example, the LVG signal was nearly 2 times larger in terms of pyrogram peak area in Douglas fir wood compared to silver fir and spruce. This result explained why Douglas fir Py-lignin was closest to the actual Klason lignin. A varying LVG content is usually attributed to either a higher content of minerals [24], which favors the formation of lower molecular weight species, or a higher crystallinity index, which favors levoglucosan [46,47], but a few tests made in the lab did not find significant differences between either cellulose crystallinity or inorganic salts content between all samples. No specific pattern was determined for the remaining carbohydrate loadings, but they were indexed for future comparison.

Acetic acid is considered a marker for hemicelluloses as they produce most of it during pyrolysis from the loss of acetyl group on the side chains. Parallelly, furfural (F6) is also considered a hemicelluloses marker [40]. In SW, both could be found in the same quadrant, indicating a higher hemicellulose content in silver fir, even though in KW, furfural was quite distant from acetic acid and close to levoglucosan, which was hard to interpret. Another marker discussed by the authors is 4-hydroxy-5,6-dihydro-(2H)-pyran-2-one, which is a marker for xylans. Its strong tendency to be around Douglas fir samples may indicate a higher amount of xylans in this species than in spruce or silver fir.

All the identified compounds separating spruce and larch from pine by Alves et al. [5], namely vanillin, cis-allenylguaiacol, trans-isoeugenol, and dihydroconiferyl alcohol, were found to separate silver fir from the other species in stemwood and knotwood.

No inter-specific loadings could be identified by comparing all the trees of a given species. These results emphasized the fact that the method does separate different species or tissues well, but the markers are heavily dependent on the species that are compared. This problem is mitigated when comparing different tissues, as presented in the next paragraph.

#### 3.2.2. Inter-Tissue Differences

The PCA results between different tissues for spruce, silver fir, and Douglas fir are also presented in Figure 4. Sp had an explained variance of 47.6% (PC1: 26.5%, PC2: 21.1%), Si of 49% (PC1: 28.7%, PC2: 20.3%), Do 57.7%: (PC1: 37.2%, PC2: 20.5%). The lower explained variances were due to the more spread-out clusters. Branchwood differentiated itself through H1, H2, H3, G1, G2, G5, G9, and G14, with those loadings being the largest pyrolysis products of both *p*-hydroxyphenyl and guaiacyl units. H units were an indicator of compression wood as it is composed of more H-lignin than stemwood [48,49]. As discussed before, lignin content is higher in compression wood, which also explains a higher presence of the naturally most prominent G-unit pyrolysis products.

Other compounds of low molecular weight, like C1, C2, C3, C4, F1, F2, and F3, were located towards the branchwood of spruce. It is known that lignin pyrolysis can produce those compounds, and for our Py-lignin estimation, we only ascribed those compounds to carbohydrate products to facilitate the method. Although all three species have around 35% lignin content in their branches, only spruce had this tendency, not silver fir or Douglas. This may indicate that they could be indicative of markers but only for this one species.

O-cresol (H2) varied between stemwood and knotwood, unlike m- and *p*-cresol (H3). Brennan et al. [23] also identified H-lignin pyrolysis products to differentiate stemwood from compression wood along with most other G lignins (apart from G14), though they treated their wood with CaCl2, which influenced the pyrolysis reaction. Although Alves et al. [5] identified propioguaiacone (G14) as a marker for compression wood in spruce, this compound did not seem to be a decisive marker of this fact, except for silver fir in our case (Si).

The sole marker that originated from carbohydrates and was more present in branchwood was 5-methylfurfural (F9). All other carbohydrate markers were mostly in stemwood or knotwood for silver fir and Douglas fir and branchwood for spruce.

Although Spruce (Sp), knotwood, and stemwood were superposed, the transitioning could be clearly seen from stemwood to knotwood in silver fir (Si) and Douglas fir (Do), where certain samples were more similar to stemwood (Si) and some more to branchwood (Do), which separated stemwood from compression wood. Comparing (BW) to (Sp-Do) showed a clear separation of branchwood from the heart- and knotwood and a certain homogeneity among themselves.

Knotwood can be considered to be an intermediary between stemwood and branchwood in terms of tissue. Though the superposition of stemwood and knotwood in silver fir and spruce made it hard to distinguish between them, some compounds mostly from the furan family, like furfural (F6) and 5-hydroxymethylfurfural (F11), seemed to separate knotwood from the other samples.

From a phylogenetic point of view, the proximity of silver fir and spruce and their distance from Douglas fir is slightly unexpected since, in the *Pinaceae* family, the *Picea* subfamily is closer to *Pseudotsuga* than *Abies* [50]. The factors differentiating polymer synthesis might be less about genetic proximity and more about environmental factors and epigenetic factors. Silver fir and spruce are indigenous to Europe, although they are planted farther out of their natural habitat, usually 400 m in altitude or Nordic countries. Douglas fir was introduced to Europe around 1820, while it is originally from the East coast of the United States up to Canada. Hintsteiner [51] compared the 13 nuSSR loci from Douglas fir to pinpoint the origin of several stands of Douglas fir across Europe and found most of them to originate from the same strand from central Washington. Since this strand is the recommended provenance for Douglas fir acorns still today, trees grown from this seed are the most likely to adapt to the European climate while not changing much, therefore remaining similar to trees that would be grown in Washington.

#### 3.2.3. Height Differences

PCAs were also attempted to determine markers of longitudinal variation. Unfortunately, they most often seemed to separate different trees rather than height. When isolating trees, results showed mostly similar samples between heights, as reflected in the PCAs presented before. Isolating specific loadings differentiating height could not be done for lignin, but some potential markers could be isolated, most notably acetic acid, which was more often representative of the top samples. This might be due to the top having a larger proportion of juvenile wood, which contains more hemicellulose, which is the largest contributor of acetic acid in pyrolysis [22,32,52]. A larger presence of hemicellulose might mean less lignin and cellulose, and as such, levoglucosan was often found at the bottom samples, as well as F6 and F11. Those being large products, they are easier to identify on PCAs.

## 4. Conclusions

Py-lignin was confirmed to be able to correctly describe the variation of lignin according to height along the stem, knots, and branches of spruce, silver fir, and Douglas fir. A correction factor must be applied, taking into account the wood species and the wood compartment that is being studied, highlighting that Py-lignin needs to be defined for every tissue and species before replacing the Klason lignin method. Differences along the branches were numerous and related to compression wood presence. Its presence induced a greater difference in lignin content between two neighboring samples compared to the whole variation of the stemwood or knotwood. The Py-GC/MS appears to be an interesting method for a semi-quantitative evaluation of lignin content in wood.

Polysaccharide variation was also studied along the stem height. Statistical analysis allowed a conclusion that no significant difference existed in the content of the different biopolymers according to height in all three studied species, with only small differences being observed.

Multiple pyrolysis markers could be identified to separate tree species (levoglucosan separates Douglas fir from spruce and silver fir), different tissues (Branchwood differentiates itself mostly from H-lignin as well as 5-methylfurfural), and finally, height (more acetic acid in the “top” samples) which could be of great interest for further studies dealing with wood chemical variability.

The presence of H unit markers helps detect the presence of compression wood, its abundance increasing from stemwood to knotwood and from knotwood to branchwood.

## Figures and Tables

**Table 1:** Height of each of the stemwood and knotwood samples within the tree, stand management, and tree characteristics. Heights are given in cm above the ground: MILAB, mid-height between stem base and DEC20AB; H1BVAB, height of the first green branch; HBHAB, height of the base of the crown; DEC20AB, height at which the stem is 20 cm in diameter; DEC10AB, height at which the stem is 10 cm in diameter. DBH, diameter at breast height, measured in cm. Samples taken at breast height (130 cm) are not included.

Species	Management	Tree	MILAB	H1BVAB	HBHAB	DEC20AB	DEC10AB	Height	Age	DBH
*Abies alba*	Un-thinned	1	480	1130	1365	1685	1705	2240	50	28
	Un-thinned	2	330	1136	1350	-	617	1575	44	13.9
	Thinned	3	697	495	795	1350	1745	2140	43	41.2
	Thinned	4	685	703	890	1318	1720	2120	44	38.9
*Picea abies*	Un-thinned	1	1040	1540	1965	2036	2567	3065	53	40.9
	Un-thinned	2	860	1470	868	376	1595	2203	53	22.3
	Thinned	3	836	915	1295	1710	2250	2693	53	39.3
	Thinned	4	-	932	1414	1857	2396	2844	53	40.9
*Pseudotsuga menziesii*	Un-thinned	1	1103	1882	2059	2211	2716	3175	48	47.7
	Un-thinned	2	-	1767	1918	1512	2452	3120	48	31.4
	Thinned	3	-	1112	1421	2142	2590	3072	48	31.4
	Thinned	4	925	810	1065	1880	2200	2655	48	52.5

**Table 2:** Retention times (RT) of identified compounds used for Py-lignin determination. The labels are as follows: carbohydrate (C), furan (F), anhydrosugar (A), *p*-hydroxyphenyl lignin (H), guaiacyl lignin (G). The area percent range includes all the samples.

No	RT	Compound	Main Fragments (*m*/*z*)	Label	Area Percent Range (%)
1	4.42	Acetaldehyde	29, 43, 44	C1	1.34–3.46
2	4.77	Furan	39, 68	F1	0.16–0.64
3	4.97	2-Propenal	27, 55, 56	C2	1.06–3.13
4	5.14	Propanal-2-one	43, 72	C3	4.36–8.92
5	5.73	2-Methylfurane	82, 81, 53	F2	0.29–1.17
6	6.25	2-Butenone	55, 70	C4	1.06–2.33
	6.29	2,3-Butanedione	43, 86		
	6.34	1,3-Dimethoxypropane	45, 72		
7	6.61	3-Pentanone	29, 57	C5	0.53–1.55
8	7.35	Hydroxyacetaldehyde	31, 60	C6	2.96–10.37
9	7.60	2,5-Dimethylfurane	95, 96	F3	0.08–0.27
10	8.04	2-Butanol	43, 45	C7	0.02–0.52
11	8.34	Acetic Acid	43, 45, 60	Acetic acid	1.16–5.52
12	8.67	2,3-Pentanedione	43, 57, 100	C8	0.11–0.54
13	9.26	Ethylpropylether	29, 58, 86	C9	0.16–0.60
14	9.35	1-Hydroxy-2-propanone	43, 74	C10	2.11–6.33
15	10.08	Methyl formate	31, 60	C11	0.27–1.56
16	12.46	1,2-Ethanediolmonoacetate	43, 73, 74	C12	0.61–4.78
17	12.90	Unknown furan compound	55, 84	F4	0.09–0.61
18	13.50	2-(5H)furanone	54, 84	F5	0.33–1.07
19	13.86	Unknown compound	43	C13	1.66–6.76
20	14.39	Furfural	95, 96	F6	0.93–3.38
21	15.64	Unknown lactone derivative	55, 98, 105	C14	0.09–0.27
22	15.79	Furfuryl alcohol	69, 81, 98	F7	0.06–0.37
23	15.87	1-(Acetyloxy)-2-propanone	43, 86, 116	C15	0.24–0.93
24	16.13	Unknown compound	43, 72, 96	C16	0.27–0.89
25	16.57	Acetylfuran	95, 110	F8	0.05–0.20
26	17.84	2-Hydroxycyclopent-2-en-1-one	98	C17	1.17–2.75
27	18.44	5-Methylfurfural	55, 83, 112	F9	0.08–0.59
28	19.90	4-Hydroxy-5,6-dihydropyran-(2H)-2-one	58, 114	C18	0.63–3.60
29	20.48	3-Methyl-1,2-cyclopentanedione	83, 98, 112	C19	0.18–1.88
30	20.80	Unknown compound	112, 128	C20	0.12–0.25
31	21.19	Phenol	65, 66, 94	H1	0.14–1.14
32	21.65	Guaiacol	81, 109, 124	G1	1.73–5.78
33	22.37	*o*-Cresol	107, 108	H2	0.15–0.49
34	23.44	*m*-Cresol + *p*-Cresol	107, 108	H3	0.12–1.15
35	23.74	Furanic compound	85, 148	F10	0.13–1.02
36	24.16	Creosol	123, 138	G2	3.71–7.65
37	24.34	2,5-Dimethylphenol	107, 122	H4	0.21–1.27
38	24.36	Unknown compound	44, 57	C21	0.15–2.60
39	25.62	Isomer of 4-Methoxy-2,6-dimethylphenol	109, 137, 152	G3	0.12–0.39
40	26.08	Ethylguaiacol	122, 137, 152	G4	0.97–1.76
41	26.34	Unknown Anhydrosugar	69	A1	0.18–4.36
42	26.63	Unknown Anhydrosugar	71, 98	A2	0.16–0.50
43	26.76	Unknown Anhydrosugar	71, 97	A3	0.12–0.46
44	27.07	Unknown Anhydrosugar	69, 144	A4	0.13–0.31
45	27.34	Unknown Anhydrosugar	57, 91, 120	A5	0.14–0.96
46	27.39	Vinylguaiacol	135, 150	G5	3.92–7.11
47	27.88	Eugenol	149, 164	G6	1.35–2.51
48	27.93	Propylguaiacol	137, 166	G7	0.14–0.94
49	28.42	5-Hydroxymethylfurfural	69, 97, 126	F11	0.07–1.37
50	29.09	Cis-isoeugenol	149, 164	G8	0.90–1.67
51	29.70	Allylphenol	133, 134	H5	0.09–1.03
52	30.02	Unknown Anhydrosugar	87, 144	A6	0.05–2.27
53	30.18	Trans-isoeugenol	164	G9	4.32–7.64
54	30.81	Vanillin	151, 152	G10	1.57–3.22
55	31.01	*Cis*-allenylguaiacol	147, 162	G11	0.13–0.50
56	31.23	*Trans*-allenylguaiacol	147, 162	G12	0.17–0.48
57	31.92	Homovanillin	137, 166	G13	0.91–2.46
58	32.25	Propioguaiacone	151, 180	G14	0.15–0.74
59	32.41	Acetoguaiacone	151, 166	G15	1.03–1.90
60	33.32	Guaiacylacetone	122, 137, 180	G16	0.61–2.44
61	34.20	1′-Hydroxyeugenol	137, 180	G17	0.74–1.29
62	34.37	Vanilloylacetyl	123, 151, 194	G18	0.08–0.30
63	35.74	Levoglucosan	60, 73	LVG	1.76–11.53
64	35.76	Dihydroconiferyl alcohol	137, 182	G19	0.02–0.62
65	36.39	*Cis*-coniferyl alcohol	137, 180	G20	0.01–0.30
66	37.32	Unknown Anhydrosugar	69, 73, 115	A7	0.02–0.72
67	38.07	Coniferaldehyde	135, 178	G21	0.07–2.24

**Table 3:** Chemical composition of stemwood and knotwood of the three species. Results are presented in % of extractive-free and oven-dried samples. Errors presented are in % and show variability of biopolymers between 4 trees.

	Species	Klason Lignin			Holocellulose			α-Cellulose		
		Bottom	Middle	Top	Bottom	Middle	Top	Bottom	Middle	Top
Stem- wood	*Picea* *abies*	28.1 ± 1.2	26.6 ± 0.6	26.9 ± 0.5	72.7 ± 4.3	72.9 ± 0.8	75.7 ± 1.1	41.9 ± 4.0	41.6 ± 1.1	44.8 ± 1.3
	*Abies* *alba*	29.9 ± 0.8	29.5 ± 1.3	31.0 ± 2.6	73.2 ± 2.1	74.5 ± 3.9	71.3 ± 3.9	42.2 4.6	43.1 ± 5.3	34.9 ± 5.6
	*Pseudotsuga* *menziesii*	27.6 ± 1.6	29.3 ± 1.2	29.5 ± 2.0	72.3 ± 1.5	70.5 ± 2.9	69.5 ± 1.2	41.0 ± 1.5	35.3 ± 5.5	37.9 ± 2.2
Knotwood	*Picea* *abies*	32.0 ± 1.5	31.9 ± 0.9	30.1 ± 1.0	69.0 ± 6.9	72.7 ± 4.7	70.9 ± 2.5	34.0 ± 6.3	37.7 ± 2.5	36.8 ± 2.0
	*Abies* *alba*	30.6 ± 1.2	30.9 ± 0.3	30.5 ± 0.6	73.7 ± 2.7	73.6 ± 0.8	72.3 ± 2.7	38.2 ± 1.8	37.5 ± 2.2	37.2 ± 3.3
	*Pseudotsuga* *menziesii*	30.7 ± 1.4	30.5 ± 2.4	29.9 ± 2.0	65.4 ± 1.6	65.1 ± 2.5	67.3 ± 2.4	36.1 ± 1.9	36.5 ± 4.0	38.3 ± 1.7

**Table 4:** *p*-values for the ANOVA tests comparing different heights and trees of each species according to their wood compartment.

Tissue	Biopolymer	Species	Height		Trees			
			Height Interaction	*p*-Value	Tree Interaction	*p*-Value	Tree Interaction	*p*-Value
Stemwood	Klason Lignin	*Picea* *abies*	Top–middle	0.131	1–2	<0.001	2–3	<0.001
			Middle–bottom	0.103	1–3	0.006	2–4	<0.001
			Top–bottom	0.989	1–4	0.481	3–4	0.079
		*Abies* *alba*	Top–middle	<0.001	1–2	0.649	2–3	0.028
			Middle–bottom	0.207	1–3	0.205	2–4	0.086
			Top–bottom	0.006	1–4	0.011	3–4	<0.001
		*Pseudotsuga* *menziesii*	Top–middle	0.059	1–2	<0.001	2–3	<0.001
			Middle–bottom	0.943	1–3	0.588	2–4	0.102
			Top–bottom	0.033	1–4	<0.001	3–4	<0.001
	Cellulose	*Picea* *abies*	Top–middle	0.047	1–2	NA	2–3	NA
			Middle–bottom	0.444	1–3	0.942	2–4	NA
			Top–bottom	0.314	1–4	0.622	3–4	0.439
		*Abies* *alba*	Top–middle	0.001	1–2	0.325	2–3	0.064
			Middle–bottom	0.881	1–3	0.733	2–4	0.005
			Top–bottom	0.003	1–4	0.118	3–4	0.508
		*Pseudotsuga* *menziesii*	Top–middle	0.062	1–2	0.178	2–3	0.807
			Middle–bottom	<0.001	1–3	0.041	2–4	0.694
			Top–bottom	0.082	1–4	0.028	3–4	0.996
Knotwood	Klason Lignin	*Picea* *abies*	Top–middle	0.001	1–2	0.002	2–3	0.898
			Middle–bottom	0.978	1–3	<0.001	2–4	0.177
			Top–bottom	<0.001	1–4	0.101	3–4	0.056
		*abies* *alba*	Top–middle	0.104	1–2	0.105	2–3	0.359
			Middle–bottom	0.589	1–3	0.005	2–4	0.984
			Top–bottom	0.456	1–4	0.058	3–4	0.544
		*Pseudotsuga* *menziesii*	Top–middle	0.059	1–2	<0.001	2–3	<0.001
			Middle–bottom	0.993	1–3	0.588	2–4	0.102
			Top–bottom	0.033	1–4	<0.001	3–4	<0.001
	Cellulose	*Picea* *abies*	Top–middle	0.297	1–2	<0.001	2–3	0.819
			Middle–bottom	<0.001	1–3	<0.001	2–4	0.909
			Top–bottom	0.003	1–4	<0.001	3–4	0.996
		*Abies* *alba*	Top–middle	0.964	1–2	0.579	2–3	0.997
			Middle–bottom	0.277	1–3	0.684	2–4	0.469
			Top–bottom	0.189	1–4	0.997	3–4	0.571
		*Pseudotsuga* *menziesii*	Top–middle	0.336	1–2	0.594	2–3	0.921
			Middle–bottom	0.969	1–3	0.277	2–4	0.299
			Top–bottom	0.241	1–4	0.938	3–4	0.114

**Table 5:** Loadings present at different species in PCA SW-KW.

Douglas fir	C1, C2, C3, C6, C8, C11, C18, F1, F2, F5, F9, F11, A1, A2, A4, A5, A6, LVG, H3, H4, H5, G2, G3, G4, G12, G13, G15, G16, G17, G18, G20
Silver fir	C5, Acetic acid, C9, C12, C13, C16, C17, C19, F4, F7, F8, F10, H1, H2, G5, G6, G7, G8, G9, G10, G11, G19, G21
Spruce	A3, A7, G1, G14

## Data Availability

The data presented in this study are openly available in http://docnum.univ-lorraine.fr/public/DDOC_T_2022_0321_HENTGES.pdf (accessed on 27 October 2023).
